# Telehealth high-intensity interval exercise and cardiometabolic health in spinal cord injury

**DOI:** 10.1186/s13063-022-06585-2

**Published:** 2022-08-04

**Authors:** Jacob Adams, Byron Lai, James Rimmer, Danielle Powell, Ceren Yarar-Fisher, Robert A. Oster, Gordon Fisher

**Affiliations:** 1 Department of Human Studies, University of Alabama at Birmingham, Education Building, 205 901 13th St. South, Birmingham, AL 35294 USA; 2 Department of Pediatrics, Birmingham, USA; 3 Dean’s Office, Birmingham, USA; 4 Department of Physical Medicine and Rehabilitation, Birmingham, USA; 5 Department of Medicine, University of Alabama at Birmingham, Birmingham, USA

**Keywords:** Exercise, SCI, Spinal cord Injury, HIIT, High-intensity interval training, Resistance training, Upper-body exercise, Randomized controlled trial

## Abstract

**Background:**

The number of exercise trials examining cardiometabolic outcomes in spinal cord injury (SCI) is low, and prescribed exercise is often inconvenient for individuals with SCI to perform within their community. Individuals with SCI experience a myriad of barriers to exercise participation, which can include a lack of time, accessible or usable equipment and facilities, and transportation. Thus, it is imperative to identify effective modes of exercise that provide the greatest overall health benefits but do not require a significant time commitment. Low-volume high intensity interval training (HIIT) has demonstrated the same improvements in cardiometabolic health as moderate intensity exercise training (MIT), despite only requiring 20% of the total time commitment in adults without disabilities and more recently in individuals with SCI.

**Objectives:**

The primary purpose of this study is to integrate a 16 week home-based telehealth HIIT arm crank exercise training program in individuals with SCI and assess changes in cardiometabolic health.

**Methods:**

Men and women between the ages of 19 and 60 with a confirmed diagnosis of SCI between C7 and T12 will be recruited for this study. Participants will be randomized to 16 weeks of telehealth HIIT exercise two days per week or a no-exercise control group. Aerobic capacity, muscular strength, blood lipids, glucose tolerance, resting energy expenditure, blood pressure, and body composition will be assessed at baseline and 16 weeks post-training.

**Discussion:**

Inactivity associated with SCI leads to chronic cardiometabolic health conditions. The majority of exercise interventions to date show that exercise is capable of increasing physical function, aerobic capacity, and muscle mass, and strength. Additionally, we have recently shown the ability of HIIT to improve blood lipid and glucose concentrations. Advances in telehealth exercise approaches have improved the capability to prescribe home-based exercise programs. Therefore, we hypothesize that the utilization of a home-based telehealth HIIT program will improve cardiometabolic health markers, yield high adherence (> 75%), and will be more enjoyable in individuals with SCI.

**Trial registration:**

Telehealth High-Intensity Interval Exercise and Cardiometabolic Health in Spinal Cord Injury NCT04940598

## Background/Aims

Spinal cord injury (SCI) has been reported to affect over two million people in the world, with over 350,000 of those living in the United States and nearly 18,000 new cases a year [1, 2]. The life expectancy has increased in recent decades, with the average age now at 43 for individuals living with SCI [2]. This increase in age has resulted in individuals with SCI becoming more susceptible to many of the same chronic diseases associated with aging as people without disabilities [3]. One major factor for those affected by SCI is the sedentary lifestyle created by the condition [4, 5]. People with SCI have reduced mobility due to loss of function resulting from their injury. This leads to loss of muscle mass and a sedentary lifestyle which leads to a downward trend in health and function [6]. These changes have been shown to lead to substantial life limitations in employment, social availability, and normal daily activities [7]. Following rehabilitation, these limitations often lead to secondary cardiometabolic health conditions which include obesity, type-2 diabetes, insulin resistance, muscle atrophy, lower fitness levels, and pain [3, 5, 6, 8–11]. Given these health trajectories, it is critical to identify behavioral strategies to reduce or prevent these adverse conditions following a spinal cord injury.

Exercise training has been clinically proven to prevent and in many instances reverse health conditions associated with cardiometabolic diseases. However, it remains difficult for exercise physiologists and healthcare professionals to convince people without disabilities to adhere to current exercise recommendation of at least 150 minutes/week of moderate-intensity or 75 minutes/week of vigorous-intensity aerobic exercise. The primary reason for not participating in regular exercise in people without disabilities is perceived ‘lack of time’ [12]. Individuals with SCI face even greater challenges as they have few, if any options to participate in physical activity. Three of the most frequently reported barriers to participation among people with SCI are structural (accessibility to exercise facilities, exercise equipment), time-related (getting dressed/undressed, unreliable transportation services), and lack of knowledgeable exercise personnel [13].

One mode of training that may overcome barriers associated with time commitments is low volume high intensity interval training (HIIT). A growing body of evidence in people without disabilities by us [14] and others [15–18] has demonstrated the potential for low volume high-intensity interval training (HIIT) to provide comparable or superior improvements in cardiometabolic health outcomes compared to continuous moderate intensity training (MIT) that requires 60-80% greater time commitment. To date, there have not been any well-powered long-term studies assessing the effects of low volume HIIT for improving cardiometabolic health in individuals with SCI. However, in a recent pilot study [19] by our group we found that six weeks of HIIT (participants performed a total of 20 minutes of exercise consisting of 4 minutes of arm crank exercise at 25% of heart rate reserve determined from a VO2 peak test, followed by 30 seconds at 50% of peak power obtained from an arm crank Wingate Test two times per week) or MIT (30 min of continuous arm crank exercise at 55% of VO2peak as determined by a baseline VO2 peak assessment three times per week) significantly improved insulin sensitivity (9%), cardiovascular fitness (12.2%), and muscular strength (~15%) in individuals with SCI. These data demonstrate that as few as two days per week of HIIT may improve cardiometabolic health markers similar to individuals performing MIT exercise for double the weekly time commitment.

Several studies [20, 21] have reported increased exercise participation in individuals with SCI by offering telerehabilitation services that allow rehabilitation sessions to occur from home via video technologies. These new telehealth technologies have the potential to increase participation by reducing many of the barriers that impact participation in physical activity and exercise in individuals with SCI. Thus, the primary purpose of this study is to implement a home-based telehealth HIIT arm crank exercise training program among individuals with SCI and assess changes in body composition, cardiovascular fitness, muscular strength, glucose tolerance, blood lipids, and vascular health. The secondary purpose of this study is to explore the uptake and implementation of HIIT in SCI.

## Research methods and design

### Study design and participants

This is a 16-week randomized controlled trial involving a cohort of individuals with chronic SCI. Participants will be screened to determine if they meet the inclusion and exclusion criteria as outlined in Table 1.

**Table 1:** Eligibility criteria

Inclusion Criteria	Exclusion Criteria
1. Men and women, 19-60 years of age. 2. Confirmed diagnosis of traumatic SCI at the cervical or thoracic level (C7-T12), classified as A, B, C, or D (motor and sensory complete or incomplete) on the AIS scale. 3. At least 3 years post-injury. 4. Able to independently operate an arm ergometer. 5. Have access to a wireless internet connection. 6. Medically stable, able to provide informed consent.	1. Cardiovascular or renal diseases. 2. Pregnant women 3.Orthopedic conditions that prevents arm ergometry 4. Upper extremity musculoskeletal conditions that prevents arm ergometry. 5. Neurological disorder that prevents arm ergometry 6. Participation in a structured exercise program currently or in the past 3 months. 7. Unable to perform exercise interventions

The first visit includes an informed consent form, given by the graduate research assistant or the PI, Dual-energy X-ray absorptiometry (DXA) scan, blood pressure, and resting energy expenditure (REE). The second visit will be conducted at the clinical research unit (CRU) at University of Alabama at Birmingham Hospital to perform an oral glucose tolerance test (OGTT). The third visit will include a peak oxygen uptake test (VO_2peark_), Wingate arm cycle test, and a series of strength assessments. Participants will take three surveys during the preliminary testing: the SF-36v2 survey, Spinal Cord Independence Measure (SCIM) survey, and a general life satisfaction survey. The first and third visits will be performed at the Lakeshore Foundation in Birmingham, AL. Participants will be randomly assigned to a no-exercise control group or an HIIT exercise group based on the block randomization method. This will be administered via closed envelopes by the PI or GRA after the preliminary testing is completed. Individuals in the control group will continue their normal daily activity but not engage in physical, planned or structured exercise during the duration of the study. Participants randomized to the exercise group will perform home-based arm crank HIIT exercise 2 days a week for 16 weeks. After 16 weeks participants will return and repeat the baseline assessments.

### Study timeline

**Fig. 1:**
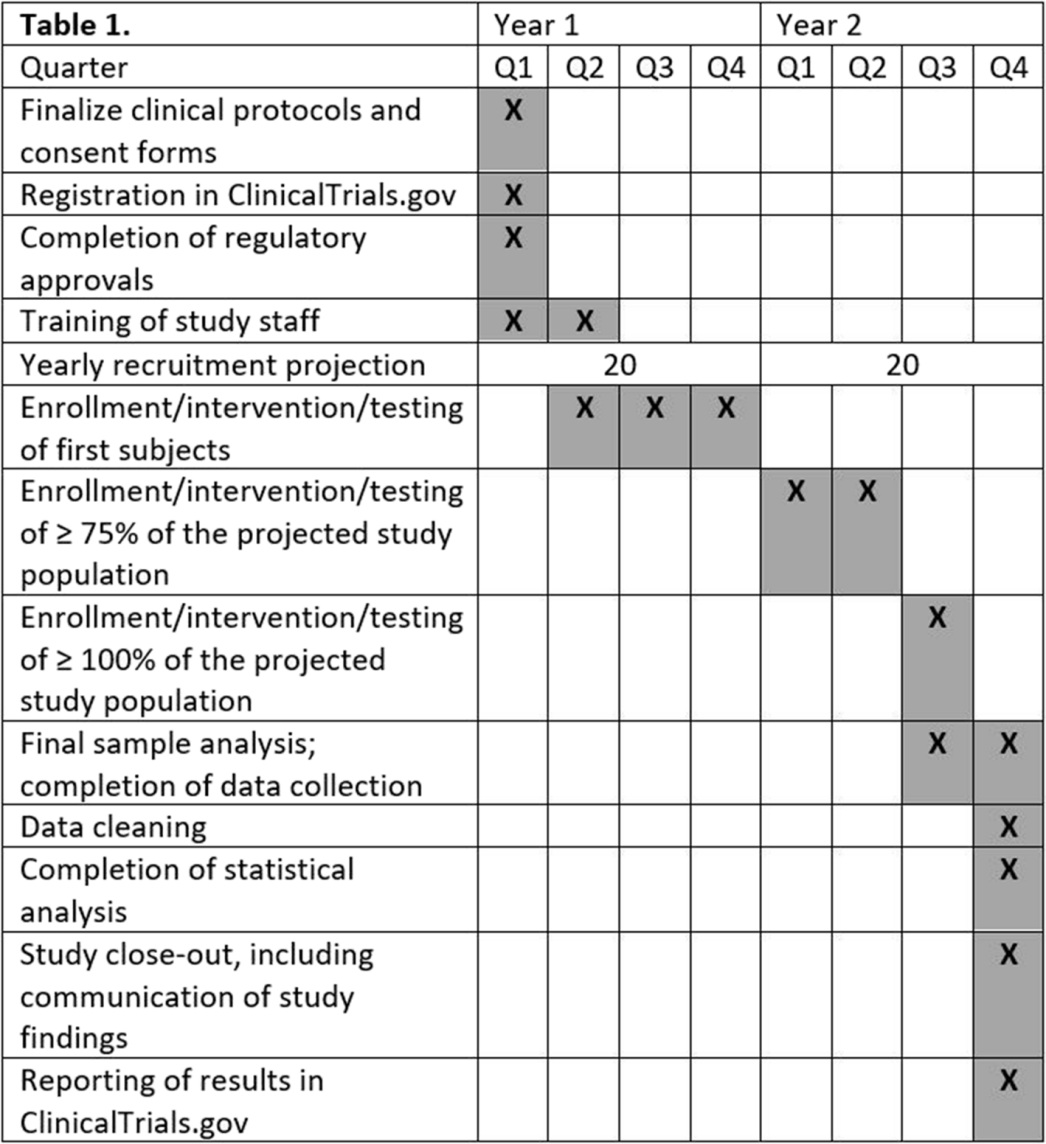
Study Timeline shows the projected two-year timeline of this research study. ^#^: We intend to recruit 40 people while accounting for a 90% participation rate (n=36 enrolled, n=4 drop out)

**Fig. 2:**
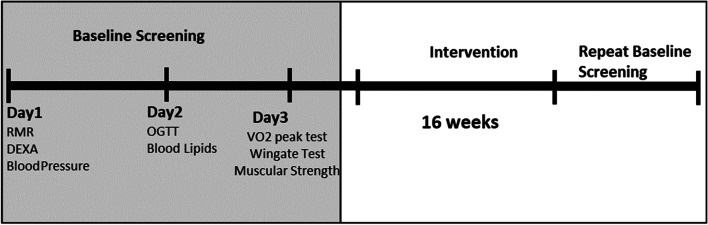
Participant Timeline Prescribed timeline of a participant’s involvement in this research study. *Note*. Prescribed timeline of a participant’s involvement in this research study

The study timeline is outlined in Fig. 1 and the testing schematic is outline in Fig. 2.

## Recruitment

Participants will be recruited from a large SCI national longitudinal database. Potential subjects will be initially contacted via phone and given an in-depth description of the study, those interested will be followed up with via email with an inclusion listing. Those that complete the preliminary testing and the post testing after 16 weeks will be monetarily compensated for their time. Participants will be compensated $100 after randomization and then $200 after completion of the study.

## Groups

### No-exercise control group

Participants randomly assigned to the no-exercise control group will undergo normal daily activities and will be asked to refrain from participating in structured exercise.

### Exercise group

The **teleexercise** will be delivered through a custom, wireless internet-based system that will be installed in the participant’s home. The equipment within this system includes a tablet computer (Samsung Galaxy Tab 2 10.1, Samsung) with Bluetooth® and wireless Internet capability mounted to an adjustable floor stand (Standzfree Universal Stand, Standzout); wearable physiologic monitor (Bioharness 3, Zephyr) that provides real-time monitoring of heart and respiration rate data to the tablet via Bluetooth® connection; and custom-designed web application that allows physiologic data and video feed to be recorded from the tablet to a secure web-based dedicated server. This platform allows the exercise trainer (telecoach) to monitor each participant’s physiologic data in real-time (up to 5 second delay) while simultaneously video-conferencing and providing written instructions to the participant. Telecoaches will utilize this system to provide immediate feedback regarding exercise intensity and movement quality during each exercise session. All exercise sessions will be performed on an upper body ergometer (UBE-BDP Table Top Upperbody Exerciser, Hudson Fitness).

**HIIT training** will be delivered two times per week for 16 weeks (32 sessions). Each session will be separated by at least 24-hrs. Participants will be allowed to choose the days and times that works best for their schedule. The HIIT protocol will be determined based on peak anaerobic power measures during an arm crank Wingate Cycle test. HIIT will consist of 20 minutes of continuous exercise consisting of four minutes of arm crank exercise at 5% of peak anaerobic power followed by 30 seconds at 30% of the peak anaerobic power; this cycle will be repeated four times, ending with two minutes of recovery at 5% of peak anaerobic power. The use of percent workload is important as use of heart rate to prescribe exercise is not accurate in many individuals with SCI, as the sympathetic nervous system limits heart rate response to exercise in people with T5 or higher lesions [22]. Based on our preliminary data in which some participants had difficulty performing intervals using 50% of peak anaerobic power, this protocol will utilize a more modest intensity of 30% of peak anaerobic power, which should be more feasible and achievable for all participants. This workload will still be higher than corresponding VO_2_ peak workloads, thus will still elicit a high intensity interval session. The participants will be coached and monitored remotely via the telehealth system. Heart rate and respiratory rate will be monitored throughout each session.

## Assessments

### Dual-energy X-ray Absorptiometry (DXA)

The total body composition will be measured in sections using DXA (Lunar Radiation Corp, Madison WI) to determine the fat mass, lean mass, and bone mineral density in the participant’s body. This data will be presented as a whole, as well as specified anatomical regions (arms, legs and trunk).

### Resting Energy Expenditure (REE)

The REE will be measured following a 12 hour fast. This will be measured with the participant laying supine on a bed with a plexiglass enclosure around the head. The participant will undergo a 15-minute rest period before testing begins. The test will include a 30-minute measurement period using an open circuit, computerized, indirect calorimetry system connected to the plexiglass enclosure placed over the participant’s head.

### Oral Glucose Tolerance Test (OGTT)

The OGTT will be performed by trained nurses and physicians at a large medical hospital. This test will be administered following a 12 hour fast. The subject will consume 75g of glucose orally within a 5-minute time frame. Blood samples will then be collected after 60, 90 and 120 minutes from when the glucose was ingested.

### Serum analysis

All serum obtained from the OGTT (glucose, insulin, HDL, LDL, triglycerides and cholesterol) will be analyzed to determine concentrations of each factor.

### Blood pressure

The subject’s blood pressure will be taken using both an automated blood pressure cuff and manual cuff testing performed by a trained researcher. Blood pressure will be taken from the left arm while the arm is rested at approximately 145 degrees.

### Peak Oxygen Uptake (VO_2peak_)

All subjects will undergo a progressive peak oxygen assessment to determine aerobic capacity. Participants will be instructed to use an arm crank ergometer (Lode) at 10W for 2 min. Every 2 min thereafter, power output will be increased by 10W until voluntary fatigue. Peak aerobic power will be defined as VO2 at the point of failure to maintain 60-65 rotations per minute (rpm) [23]. Minute ventilation, oxygen uptake and carbon dioxide production will be continuously analyzed and recorded by an open-circuit spirometry system (ParvoMedics). Heart rate will be continuously assessed using a polar heart rate monitor. Rate of perceived exertion (RPE) during exercise will be assessed using the BORG scale.

### Wingate test

All subjects will perform the Wingate Anaerobic Power test [24]. Subjects will sit in a chair (fixed to the ground) and remain seated throughout the entire test. The seat height and back will be adjusted so that the crank position on the opposite side to the body and the hand grasping the handles, allowing the elbow joint to almost fully extend (165-175°) and the shoulders in line with the center of the ergometer’s shaft. A fly wheel braking force corresponding the 5% of the participants body weight will be used. Prior to each test, participants will complete a 5-minute warm up at 10W, which will include 3 short sprint efforts followed by a 5 min recovery period. Following the warm up participants will be instructed to hand cycle as fast as possible, verbal encouragement will be given to all participants to maintain their highest possible cadence throughout the test. Peak power, mean anaerobic power, fatigue rate, and relative peak power will be assessed following each test. The physiological data obtained from this test will be used to calculate exercise intensity corresponding to 30% of peak power for the exercise intervals.

### Muscle strength assessment

A muscle strength assessment will be performed to determine participant strength measurements. This will include arm curls, wheelchair pushups, and hand grip strength.

Arm curls will be assessed using a 5-25lb weight determined by age, gender and body weight. Participants will perform the arm curl from a seated position to the beat of a metronome until the arm becomes fatigued. This test will be performed on both arms, starting with the participant’s dominant arm.

Wheelchair seated pushups will be conducted by the participant pushing up on the arm rest of a chair or wheel of their chair. One repetition will include full extension so that the elbows are straight and then bends so that the buttocks returns to the chair. The participant will continue these repetitions until they are unable to continue.

Grip strength will be tested using a Jamar hand dynamometer. This test will be administered three times per hand with an additional practice run before beginning. The average of the three tests will be taken per hand.

## Statistics

Sample Size Considerations: Power calculations were performed using nQuery 8.5. We will recruit 20 participants in each group. Assuming a 10% drop-out rate, we expect 18 participants per group to complete the study. Assuming a standard deviation for VO_2_ peak of 6.0 mg/kg/min (obtained from our preliminary data) [25], 18 per group, a two-sided two-group t-test, and a 5% significance level, we will have 80% power to detect between-group differences of 5.8 mg/kg/min and greater in VO_2_ peak as being statistically significant.

### Allocation

In a longitudinal study design, 40 participants with chronic SCI will be randomly assigned to one of the two study groups (HIIT and control) in a 1:1 ratio. Randomization will be performed using the block randomization method. A randomization list will be generated and assignments will be placed into sealed envelopes and given to each study participant by the principal investigator or graduate research assistant (GRA) after the baseline testing assessments.

### Data analysis

Descriptive statistics will be obtained for all study variables of interest. Overall comparisons between means of the two groups will be performed using the two-group t-test, and overall changes within groups will be examined using the paired t-test. The primary method of analysis will be mixed models repeated measures analysis. This will allow us to test the group, time, and group by time interaction effect simultaneously. An appropriate structure for the covariance matrix (e.g., unstructured) will be selected for these models using the final data. The Tukey-Kramer multiple comparisons test will be used as the *post hoc* test of choice for these analyses. Biological variables, such as age, sex, race, and % fat, will be accounted for in these models. Pearson or Spearman correlation analysis, as appropriate, will be performed. Distributions of continuous variables will be examined for normality using box plots, stem-and-leaf plots, normal probability plots, and the Kolmogorov-Smirnov test; those deviating greatly from a normal distribution will be log transformed or analyzed using appropriate nonparametric tests such as the Wilcoxon rank-sum and signed-rank tests. Multiple imputation methods may be used to address missing data. Statistical tests will be two-sided and will be performed using a 5% significance level. SAS software, version 9.4 or later, will be used to conduct the statistical analyses.

The feasibility component of this project will be evaluated via mixed-methods, framed within dialectical pluralism: a meta-paradigm or viewpoint that calls for the use of both quantitative and qualitative belief systems when implementing a mixed-methods study [26]. Accordingly, separate theoretical assumptions will be held for each study component (Quantitative: Positivism/Postpositivism; Qualitative: Interpretivism) and equal value will be placed on both the quantitative and qualitative methods and analyses. Evaluation of feasibility will include an integrative evaluation of both quantitative and qualitative feasibility outcomes by the key members of the research team. When possible, quantitative outcomes will include a priori benchmarks to be classified as *acceptable*. The remaining outcomes will be descriptively analyzed and discussed.

At post-intervention, participants will undergo a semi-structured interview to describe factors that affected their adherence and explore their perceptions of completing the program, with special attention to *satisfaction* and *value* (core constructs of system usability testing). The semi-structured interviews will contain seven open-ended questions related to the following areas: 1) overall perceptions of the program, 2) program likes, 3) dislikes, 4) perceived satisfaction and 5) value, 6) technology and equipment usability, and 7) factors that affected adherence. These areas will be probed in greater detail by the interviewer through additional follow-up questions. The interviews will be conducted by a research member that is not involved with the exercise training. Interviews will be recorded via audio devices, transcribed verbatim, and subject to coding.

The qualitative research will be informed by ontological relativism (i.e., reality is multiple and subjective) and epistemological subjectivism (i.e., knowledge is socially constructed) [27]. Qualitative data will be analyzed by two researchers, using latent thematic analysis following the six steps proposed by Braun et al [28]. To support the quality of these data, the analysts will use two strategies, namely, member reflections and critical friends [29, 30].”

## Data and safety monitoring plan

All human participant data and laboratory test will be reviewed each quarter by the Principal Investigator (PI) as well as Co-Investigators to discuss potential new hazards, risks or adverse effects that may occur as a result of this study. If at any point within the study hazards or risks are identified that may lead to an unusually high number of adverse events, the PI will consult the investigation team as well as the Institutional Review Board (IRB) to discuss whether the study should be altered. In the event that a particular section of the study is deemed hazardous, the procedure will be removed or replaced. Data collected during each session will be periodically reviewed to potentially identify risks and hazards before they become an issue. If at any point a participant is experiencing increased risks or complications during the study, the study physician, or the participant’s normal physician, will be consulted and will determine if the participant can remain in the study or should be asked to abstain from continuing. Participants who are pregnant, experience cardiometabolic or vascular issues, renal insufficiency or liver disease will not be included, despite there being no research that shows exercise interventions will negatively affect these conditions. The Data and Safety Monitoring Committee (DMSC), consisting of the PI and two senior faculty members at UAB, will review data reports on recruitment, enrollment, retention, adverse events, safety endpoints, and adherence at each quarterly Data and Safety Monitoring meeting. In conjunction with these meetings, we will submit a report to the NIH each quarter, which summarizes the overall study progress. When the study is completed, results will be published in the scientific literature and presented at medical facilities, as well as at scientific conferences.

## Confidentiality

In order to protect the privacy of the participants, all paperwork will only contain the participants study number and will be locked in the Principal Investigator’s office. Any information saved digitally will be stored in a room safeguarded with keycard entry. Upon completion of the study, all data will be de-identified in order to remove all links to the participants. Any shared data or shared archive will not include any identifiable links. In order to ensure all data is kept securely the following will occur before any data is shared:

- Deletion of 18 HIPAA identifiers completed automatically by REDCap.
- Secure university specific based single sign-on protocols will be followed via University of Alabama at Birmingham secure login system.
- Users will have to authenticate themselves before accessing data.
- Users will only be given access to specific information set by the Principal Investigator to limit the ability to identify participants. The file containing participant personal information will be stored in a secure file by the Principal Investigator at the University of Alabama at Birmingham.

After biospecimens are analyzed, any remaining blood samples will be kept for at least three years after the study is completed, in case any measurements need to be repeated or future explorations are performed. Participants will be notified that their specimens will be stored in case of future use (both verbally and in the informed consent) and will have the opportunity to decline to participate in the study. For participants who withdraw from the study and request that their specimens be removed from the study, we will destroy all of their remaining biospecimens. Requests to destroy samples must be made in writing. All blood specimens will be disposed of following standard operating procedures in the PI’s laboratory and Human Physiology Core of UAB’s Diabetes Research Center, which involve placing specimens in biohazard bags, followed by incineration.

## Dissemination

The Principal Investigator for this study will comply with the NIH Policy on the Dissemination of NIH-Funded Clinical Trial Information in NIH Guide Notice NOT-OD-16-149. All clinical trials for this project were registered in ClinicalTrials.gov no later than July 1, 2020. The PI will be responsible for registering the trial and will ensure that information in the clinical trial record is updated at least once every 12 months and will ensure that results are reported no later than one year after the clinical trial primary completion date.

The consent form for this clinical trial contains language specifying that the study is registered at clinicaltrials.gov. The required wording on all consent forms by the University of Alabama at Birmingham (UAB) Institutional Review Board for Human Use is: A description of this clinical trial will be available on http://www.ClinicalTrials.gov, as required by U.S. Law. This website will not include information that can identify participants. At most, the website will include a summary of the results. The UAB Center for Clinical and Translational Science (CCTS) works with investigators to ensure that they meet the requirements of ClinicalTrials.gov in a timely fashion, and that all clinical trials comply with requirements for registration and reporting as specified in NOT-OD-16149.

The study design, collection, management, analysis, interpretation of data, writing of the report or the decision to submit for publications will be determined by the research team, however final decisions will be determined by the study PI.

## Discussion

People with SCI are often at greater risk for chronic disease compared to the general population including a higher risk for cardiometabolic issues [31]. SCI has been shown to lead to multiple secondary health conditions that would be preventable with the appropriate precautions. Our previous pilot study showed that exercise significantly improved insulin sensitivity, cardiovascular fitness, and muscular strength in individuals with SCI which leads to an overall increased quality of life and decreased change for chronic health conditions [19].

Previous research studies demonstrated that high-intensity interval training over short time intervals resulted in an increase in overall cardiometabolic health, as well as a decrease in blood lipids in obese populations [5, 8, 14]. In one study by Hicks et. al. involving 34 people with SCI, significant increases were obtained in overall quality of life, perceived health, strength and mobility, while also reporting a decrease in pain and stress. However, the study only focused on endurance and low to moderate intensity exercise rather than HIIT [32]. Astorino et. al., however, demonstrated that HIIT was not only preferred over MIT, but it also showed a greater increase in markers that could lead to higher cardiometabolic health and calls for additional research on interval training in participants with SCI [33]. HIIT has also been reported to show superior improvements in cardiometabolic health while only requiring as little as 30% of time commitment based on other recommended programs [14–18, 34].

Individuals with SCI report lower ability to travel, low accessibility with public gyms and a higher price of living which reduces the ability to purchase equipment [3, 21]. This reported lack of accessibility shows the need for a remote based exercise program for this population. Thus, emerging new telehealth technologies have the potential to increase participation by reducing many of the barriers that impact participation in physical activity and exercise in individuals with SCI. This study will implement a long-term exercise study (16 weeks) that enhances accessibility to the SCI population by incorporating a remote telehealth exercise platform that will allow the training to take place in the participants home during a time period that is convenient for them. Studies have shown that telehealth rehabilitation efforts have been successful in increasing participation and accessibility in the SCI population [20, 21]. To our knowledge, this is the first study to extend the training period to 16 weeks. By extending this time frame, we would expect to see greater gains in our target outcomes. For example, our recent pilot study [19] demonstrated improvements in insulin sensitivity, glucose tolerance, cardiovascular health and muscular strength in individuals with SCI in as few as 6 weeks of HIIT training. This project will expand on these findings and determine the effects of long-term training versus the previous short-term studies in order to provide additional evidence-based data that may potentially support the use of HIIT as a mode of exercise for participants with SCI.

### Trial Status

This is an ongoing trial. Recruitment began on September 22, 2020. Enrollment is expected to be completed in July 1, 2023.

## Data Availability

Yes, upon request from the corresponding author.

## Abbreviations

UAB: University of Alabama at Birmingham
SCI: Spinal cord injury
DXA: Dual-energy X-ray Absorptiometry
REE: Resting energy expenditure
PI: Principal investigator
OGTT: Oral glucose tolerance test
RMR: Resting metabolic rate
HIIT: High intensity interval training
MIT: Moderate intensity interval training
